# Podocyte injury in diabetic nephropathy: implications of angiotensin II – dependent activation of TRPC channels

**DOI:** 10.1038/srep17637

**Published:** 2015-12-10

**Authors:** Daria V. Ilatovskaya, Vladislav Levchenko, Andrea Lowing, Leonid S. Shuyskiy, Oleg Palygin, Alexander Staruschenko

**Affiliations:** 1 Department of Physiology, Medical College of Wisconsin, Milwaukee, Wisconsin 53226, USA; 2 Cardiovascular Center, Medical College of Wisconsin, Milwaukee, Wisconsin 53226, USA; 3 Institute of Cytology, Russian Academy of Sciences, St. Petersburg, Russian Federation

## Abstract

Injury to podocytes is considered a major contributor to diabetic kidney disease: their loss causes proteinuria and progressive glomerulosclerosis. Podocyte depletion may result from improper calcium handling due to abnormal activation of the calcium permeant TRPC (Transient Receptor Potential Canonical) channels. Angiotensin II (Ang II) levels are found to be elevated in diabetes; furthermore, it was reported that Ang II causes activation of TRPC6 in podocytes. We hypothesized here that Ang II-mediated calcium influx is aggravated in the podocytes under the conditions of type 1 diabetic nephropathy (DN). Diabetes was induced in the Dahl Salt-Sensitive rats by an injection of streptozotocin (STZ-SS). Eleven weeks post treatment was sufficient for the animals to develop hyperglycemia, excessive urination, weight loss, microalbuminuria, nephrinuria and display renal histological lesions typical for patients with DN. Patch-clamp electrophysiology performed on podocytes of the freshly isolated glomeruli showed enhanced basal TRPC channel activity in the STZ-SS rats, and increased response to Ang II; total calcium influx triggered by Ang II application was also augmented in podocytes of these rats. Our studies have a strong potential for advancing the understanding of TRPC-mediated effects on podocytopenia in DN initiation.

Currently about 347 million people are suffering from diabetes mellitus, and recent estimates put more than 439 million adults worldwide in danger of diabetes and its complications by 2030^1^. Diabetic nephropathy (DN) is among the most serious complications of both type 1 and type 2 diabetes, and often leads to end-stage renal disease ultimately requiring renal transplantation. The characteristic pathological changes of DN include severe albuminuria, renal hyperfiltration, glomerular basement membrane thickening, and glomerulosclerosis^2^. In the early onset of DN a dramatic decrease in the podocyte number is observed (even before the manifestation of albuminuria^3^), which results in the loss of the filtration barrier integrity, and consequent pathological changes in glomeruli permeability^4^,^5^. Podocytes are highly specialized epithelial cells located on the surface of the glomeruli capillaries, and normally prevent leakage of protein into the urine^6^,^7^. Since these cells are unable to divide, their injury and malfunction leads to proteinuria, accumulation of extracellular matrical components, and glomerulosclerosis. Additionally, podocyte depletion and structural changes are suggested to be predictors of DN progression^8^,^9^.

The mechanisms that underlie podocyte apoptosis are a focus of intense research. A novel promising target was revealed in this area several years ago: the TRPC6 (Transient Receptor Potential Canonical) calcium channel. Accumulating evidence suggested that TRPC6 channels are crucial mediators of podocyte calcium handling, and are involved in mediating glomerular permeability and maintaining the renal filtration barrier^10^,^11^. TRPC6 channels are recognized among the emerging determinants of podocyte injury. The compelling evidence supporting this view was presented when a gain-of-function mutation in TRPC6, which leads to the development of the Focal Segmental Glomerulosclerosis (FSGS), was discovered^12^. Enhanced TRPC6 expression, specifically in the podocytes, led to glomerular damage^10^,^13^. It is of particular interest that increased expression of the native TRPC6 was found in the podocytes of patients and animals with proteinuric kidney disease^13^,^14^. Therefore, it was suggested that TRPC6 causes podocyte injury via up-regulation of calcium flux which ultimately results in apoptosis^15^,^16^.

Currently, therapeutic targeting of the renin-angiotensin system (RAS) is the most validated clinical strategy for slowing the progression of DN. Ang II released into the renal interstitium is one of the key mediators of renal inflammation and fibrosis in progressive chronic nephropathies. High intrarenal Ang II levels have been definitively linked to glomeruli damage in DN^2^,^43^,^44^,^45^,^46^, whereas the fundamental effector of Ang II in the podocytes remained to be determined. TRPC channels have been associated with Ang II-induced calcium influx in many renal cell types^17^,^18^,^19^,^20^,^21^. Convincing studies reported that Ang II can aggravate albuminuria by activating TRPC6 channels in podocytes^22^, and Ang II – induced podocyte apoptosis involves altered TRPC6 expression and Ca^2+^ influx^23^. In our recent publications using knockout animal models we demonstrated the Ang II – dependent up regulation of TRPC6 channels in the podocytes of freshly isolated glomeruli at the level of single ion channel activity^20^. Our data revealed that the TRPC6 channel is responsible for the acute Ang II-activated calcium influx in the podocytes.

The current study tests the long-term effects of Ang II on TRPC6 channels in a model of type 1 diabetes induced in Dahl SS rats with a single injection of STZ (further referred to as STZ-SS)^24^. Slaughter *et al.* conducted a comprehensive analytical study and provided evidence that STZ-SS rats develop hyperfiltration, progressive proteinuria, and display renal histological lesions characteristic of those seen in patients with DN, as distinct from salt resistant Sprague Dawley or other rodent models, which have hyperglycemia, but lack essential DN features^24^. We hypothesized here that Ang II-mediated calcium influx can be aggravated in the DN podocytes, which could further lead to podocytopenia and subsequent proteinuria. The STZ-SS model in combination with unique electrophysiological approaches allowed us to measure single TRPC channel activity and live calcium concentration changes in the podocytes of the freshly isolated glomeruli. Also, for the first time we demonstrated the enhanced TRPC-mediated calcium influx in response to Ang II in the podocytes undergoing DN-related pathological changes.

## Results

### Development of diabetic nephropathy in Dahl SS rats 6 and 11 weeks after an injection of STZ

**Figure 1:**
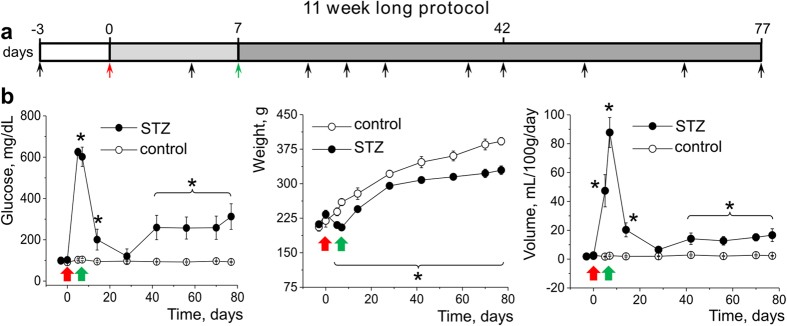
Development of STZ-induced diabetic nephropathy in Dahl SS rats during the 11 week long protocol. (**a**) Experimental protocol used for type 1 diabetes induction in the Dahl SS rat. (**b**) Development of diabetes in SS rats during 11 weeks after STZ injection as monitored by blood glucose level, body weight and urine output changes. The red and green arrows denote the time of STZ injection and subsequent insulin pellet implantation, respectively. N = 8 rats in control and 9 rats in the STZ-SS group. Asterisk denotes statistically significant difference from control values (p < 0.05).

**Figure 2:**
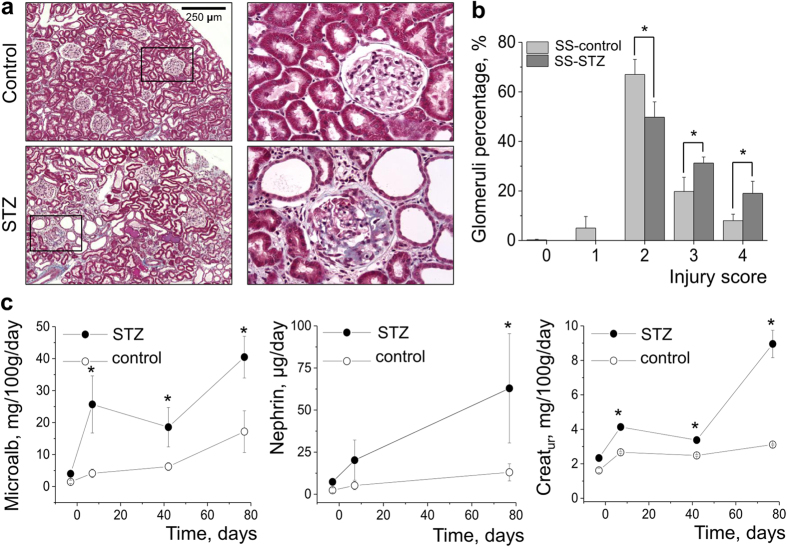
Kidney injury evaluation, microalbuminuria and nephrinuria in the course of DN development in STZ-treated SS rats. All measurements were taken during the 11 week long protocol. (**a**) Histological changes indicative of DN progression in the kidney tissue of STZ-SS rats compared to control animals. Shown are cortical fragments of the trichrome stained kidneys at 10 x and 40 x magnifications. (**b**) Glomerular injury quantification; at least 4 rats and 100 glomeruli per rat were evaluated in each group. Asterisk denotes difference from the corresponding value in control rats, p < 0.05. (**c**) Microalbuminuria, nephrinuria and urinary creatinine in STZ- SS rats versus control animals; urine samples were taken for analysis 3 days before STZ injection, on day 7 after STZ injection (day of implant administration), day 42 (except for nephrin measurement), and day 77 (terminal point). Asterisks located above the data points in the STZ-treated group denote difference from the corresponding value in control group, p < 0.05. N = 8 rats in control and 9 rats in STZ-injected group.

Induction of diabetes in Dahl SS rats (6 week old) was performed with an *i.p.* injection of streptozotocin (STZ). After hyperglycemia was confirmed (on day 5 after the injection), an insulin pellet was implanted *s.c.* and the animals were continuously monitored for 6 or 11 weeks. Fig. 1a and Supplementary Fig. 1a demonstrate the schematic representations of the 6 and 11 week long experimental protocols, featuring the days of injection, pellet implantation, and time points when urine samples were taken and blood sugar was measured. During both the 6 and 11 week protocols an initial period of severe hyperglycemia was observed within a week after the injection (up to 600 mg/mL compared to 90 mg/mL in a control group), followed by a reduction in glucose levels as a result of the insulin pellet implantation (see Fig. 1b and Supplementary Fig. 1b). Disease progression was reflected by severe polyuria and a significant decrease in the body weight of the STZ -treated rats when compared to vehicle treated rats. However, as evident from the graphs shown in Fig. 1b and Supplementary Fig. 1b , an 11 week long protocol revealed a more sustained difference in blood sugar, body weight and urine volume between the two groups. These data were confirmed by histological studies and glomeruli scoring. Fig. 2a,b and Supplementary Fig. 1c show representative images of the cortical slices of control and STZ-treated rat kidneys (terminal collection after 6 or 11 week experiments) stained with Masson’s trichrome. The 6 week long protocol did not produce kidney damage characteristic of DN (fibrosis, glomerulosclerosis, glomerular basement membrane thickening, etc), whereas 11 weeks of hyperglycemia resulted in significant glomeruli damage (Fig. 2a). To assess the degree of injury, glomeruli were scored on a scale of 0 (healthy glomerulus) to 4 (severe fibrosis and damage). Results summarized in Supplementary Fig. 1 demonstrate that 6 weeks of hyperglycemia was not enough to develop glomerular lesions compared to control (although proteinuria was developed – see Supplementary Fig. 1b), whereas an 11 week long protocol (Fig. 2b) caused an increase in the number of severely damaged cortical glomeruli.

Additionally, we tested urinary protein and electrolyte excretion during the time course of diabetes development. As reported in Fig. 2c, a stable increase in urinary microalbumin excretion was observed throughout the experiment in STZ-treated rats compared to the control group, which is indicative of impaired renal function. Furthermore, substantial urinary nephrin shedding was observed in the hyperglycemic animals (measured by an ELISA assay, Fig. 2c, middle graph); detection of nephrin in the urine is considered a solid marker of podocyte injury^25^, therefore, this finding confirms the data acquired by glomeruli scoring. Urinary creatinine levels tested throughout of the experiment (Fig. 2c (right graph)) were found to be significantly elevated in diabetic animals compared to controls, which is indicative of the damaged filtration mechanism. Sodium and potassium excretion rates (Supplementary Fig. 2) were only found to be significantly increased in STZ-treated animals compared to control animals on day 7, which should be attributed to severe polyuria developed by the animals before the insulin pellet was administered. Fractional excretion of sodium and potassium was not altered between the hyperglycemic and control groups at the terminal time point (Supplementary Fig. 2).

### Basal calcium handling is exacerbated in the podocytes during development of diabetic nephropathy

**Figure 3:**
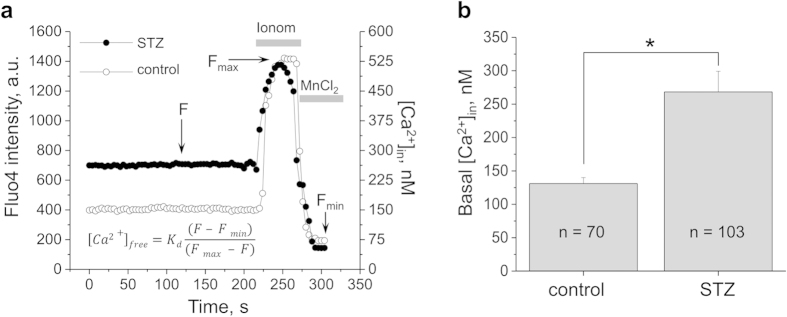
Basal intracellular calcium concentration is higher in the podocytes of the diabetic animals. (**a**) Representative calcium imaging traces showing a typical experiment designed to assess the intracellular calcium level in the podocytes. To measure intracellular calcium concentration, glomeruli were loaded with Fluo-4, AM, fluorescence intensity was recorded in the baseline and after addition of ionomycin and MnCl_2_. The graph demonstrates the fluorescence signal changes in response to ionomycin (producing the maximum of the Fluo-4,AM fluorescence, F_max_) and MnCl_2_, which quenches the dye and results in the lowest fluorescence intensity (F_min_). Intensity of fluorescence (left axis) for each time point was translated into the actual calcium concentration in nanomoles (right axis) according to the formula shown on the graph. The transients shown on the graph reflect fluorescence intensity of representative ROIs selected from glomeruli of a diabetic and control rat; images were taken every 4 s. (**b**) Bar graph summarizing the concentration of intracellular calcium in the podocytes of diabetic and control SS rats in the absence of any stimuli. Asterisk denotes statistically significant difference between groups (p < 0.05). Number of podocytes analyzed in each group (n) is shown on the graph.

On day 77 after the STZ injection, animals were sacrificed and tissues were collected for subsequent isolation of glomeruli which were then analyzed. We tested basal calcium concentration levels in the podocytes using ratiometric confocal calcium measurements. Figure 3a shows the representative traces typically used to assess intracellular calcium concentration with the help of ionomycin and MnCl_2_. Calcium levels were recorded as the intensity of Fluo-4,AM calcium binding dye, and obtained data was later recalculated into the intracellular calcium concentration according to the protocol described previously^26^. The summarizing bar graph in Fig. 3b shows a pathologically elevated calcium level in the podocytes of the STZ-treated rats (268.3 ± 30.9 compared to 131.1 ± 8.9 nM in normoglycemic animals). These data are consistent with the observed increase in basal TRPC channel activity (reported further).

### Diabetic nephropathy is accompanied with up-regulation of calcium influx in response to Ang II in the podocytes of STZ-treated SS rats

**Figure 4:**
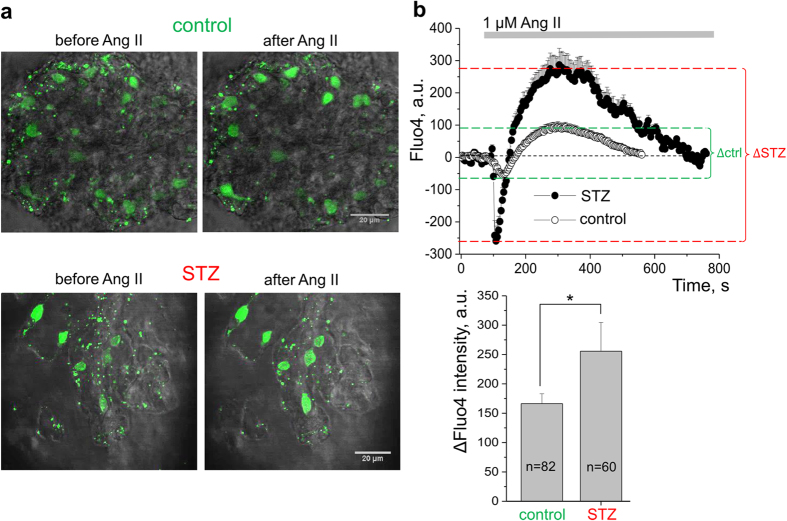
Ang II-stimulated calcium influx in the podocytes of STZ-treated and control rats. (**a**) Representative images of the Fluo-4,AM loaded glomeruli of control (upper row) and diabetic (lower row) SS rats before application of Ang II and at the point of maximal calcium influx after addition of the drug. Fluo-4,AM fluorescence intensity is positively related to calcium concentration within the podocytes. (**b**) Upper row demonstrates representative transients of intracellular calcium dynamics in the podocytes of the Fluo-4,AM loaded control and STZ-SS rat glomeruli. Low row shows the bar graph summarizing the relative changes in intracellular calcium concentration in response to Ang II in STZ-treated and control animals (reflected by Fluo-4,AM intensity). Number of podocytes for each group (n) is shown, asterisk indicates *p* < 0.05. N of rats used for these measurements was 7 in control and 6 in STZ-injected group.

Further pursuing our hypothesis, we questioned whether the increased basal calcium concentration observed in the podocytes of the STZ-treated SS rats (Fig. 3) could be due to the increased calcium influx in response to Ang II. To test this, calcium concentration changes in response to Ang II were measured in freshly isolated glomeruli of control or diabetic SS rats loaded with Fluo-4,AM. Left panel of Fig. 4 reports the typical images of the glomeruli before and after treatment with Ang II (at the peak of the fluorescence intensity). First, it should be noted that the number of podocytes on the surface of the glomeruli of STZ-treated rats was typically decreased (illustrated by the representative images), which is characteristic for the development of DN. Furthermore, Ang II induced a much higher raise in Fluo-4,AM intensity in the podocytes of the diabetic animals compared to vehicle-treated rats. As reported in Fig. 4, we observed an average 1.5 fold increase in Fluo-4,AM intensity upon stimulation with Ang II in the diabetic rat podocytes compared to controls.

### Ang II – dependent stimulation of the TRPC channel activity is enhanced in STZ-treated Dahl SS rats

As described above, basal calcium concentration and calcium influx in response to Ang II in the podocytes of the diabetic rats was significantly increased compared to the control group. We have shown earlier in our studies that the major part of the Ang II – dependent calcium influx in the podocytes is mediated via TRPC6 calcium channels^20^. To confirm our hypothesis that TRPC channels are also involved in transducing the enhanced Ang II – mediated calcium signaling in DN, we directly tested the activity of TRPC channels in our preparation. First, patch clamp electrophysiology revealed a significant increase in the number of the TRPC channels registered in each patch (from 1.25 ± 0.16 in the podocytes of control rats up to 1.9 ± 0.23 in STZ-treated animals), which aligns with the increased basal calcium levels shown in Fig. 3.

**Figure 5:**
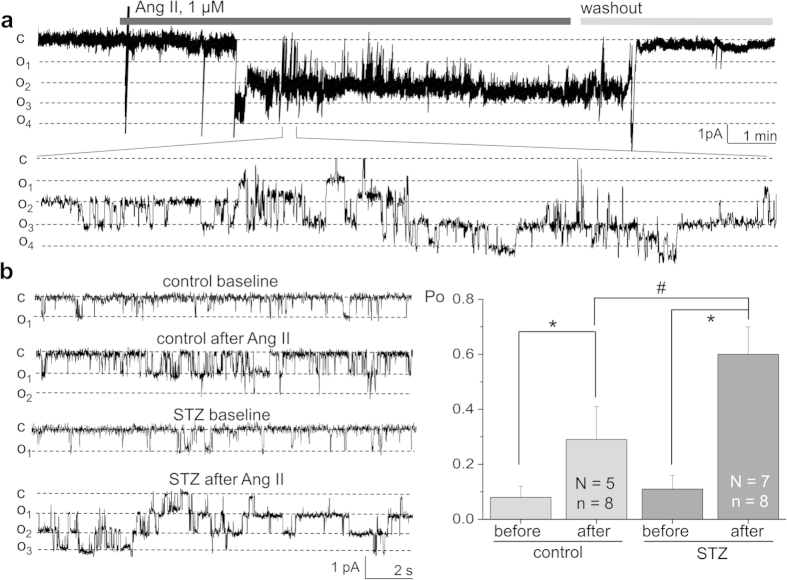
Activity of TRPC6 channels in response to Ang II in type 1 diabetes. (**a**) A representative patch-clamp recording showing the activation of TRPC channels in the podocytes of the control (vehicle-treated) SS rats in response to application of 1 μM Ang II, followed by a washout of Ang II. c and o_i_ denote closed and open states of the channel, respectively; a full recording (upper row) and a fragment of a recording at a larger scale are shown. The recording was obtained at −60 mV. (**b**) Right panel demonstrates a summary graph of the open probability (*P*_*o*_) of the TRPC channels recorded in the podocytes of the STZ-treated animals compared to control rats after acute stimulation with 1 μM of Ang II. Representative current traces illustrating TRPC channels’ activity in podocytes of diabetic rats vs. control animals (before and after application of Ang II) are shown on the left panel. N represents number of animals studied, and n is the number of analyzed patch-clamp recordings; the recordings were obtained at −60 mV. * and ^#^ denote statistical significance (P < 0.05).

Our previous studies^20^ on the wild type and TRPC6 knockout mice revealed that Ang II activated TRPC6 calcium channels in the podocytes. Here, we first confirmed that we are able to register the activity of the TRPC6 channels in rat podocytes. Recorded channels’ characteristics were similar to that of the TRPC6 channel we reported earlier in mice^20^ - reverse potential close to 0 mV, conductance of 18.6 ± 1.7 pS (data not shown). We have further shown that in the Dahl SS rats TRPC6 channels can be activated by Ang II. Fig. 5a illustrates a typical course of a patch-clamp experiment, where Ang II was applied to the bath solution and caused a fast and robust increase in the TRPC6 open probability, followed by a washout which resulted in a fast decay in the channel’s activity.

**Figure 6:**
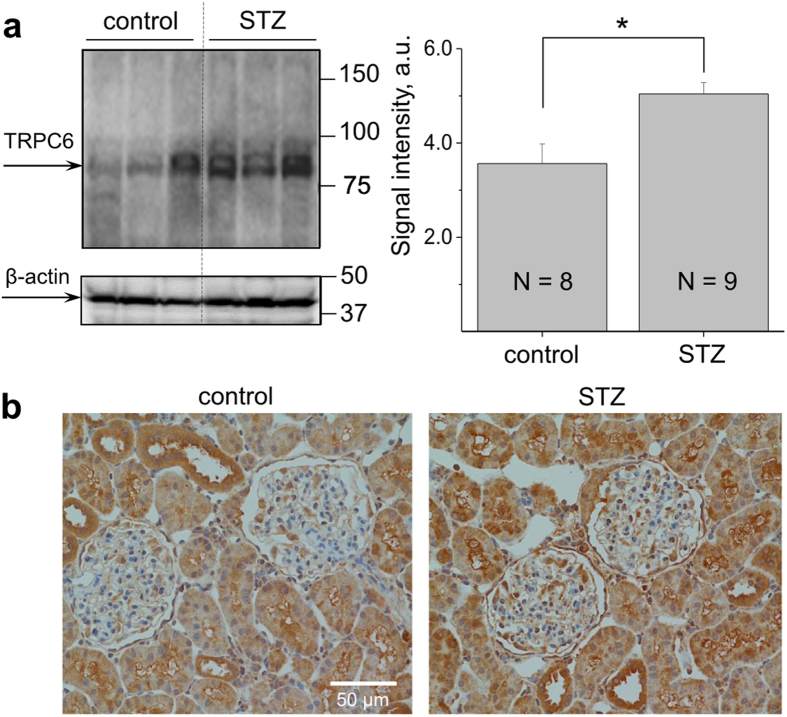
Expression levels of TRPC6 channels in the cortex of STZ-treated and control rats. (**a**) A representative Western blot and a summary graph for the densitometry values of the TRPC6 channel signal blotted from the cortical kidney lysates of the control and STZ-treated rats (terminal point, 11 weeks after STZ injection). Loading control for the representative blot (β-actin) and number of animals per group analyzed in the summary graph (N) are shown. Asterisk denotes statistical significance (P < 0.05). (**b**) Images of the representative immunohistochemical stainings for TRPC6 in the control and STZ-treated rats. Magnification – 20 x; scale bar is shown.

In our subsequent experiments we tested how the endogenous single TRPC6 channels recorded in the podocytes of the STZ-SS and control rats respond to Ang II. As seen on the current traces obtained with patch-clamp electrophysiology (see Fig. 5b, left panel), we observed a significantly enhanced response to Ang II in the podocytes of the diabetic animals compared to the normoglycemic SS rats. As reported in Fig. 5b (right panel), the increase in open probability of the TRPC6 channels in response to Ang II was substantially higher in the podocytes of the hyperglycemic rats (0.29 ± 0.12) compared to control rats (0.6 ± 0.1). Also, there was a tendency for an increase in basal TRPC6 activity in the podocytes of the diabetic rats; however, it was not significantly different from the control group. Western blotting (Fig. 6a) also demonstrated enhanced expression of TRPC6 proteins (1.45 ± 0.08 fold) in the isolated kidney cortex of the STZ-SS rats compared to control. Furthermore, the incidence of TRPC6 in patches was higher in the podocytes subject to hyperglycemia (45.5% of patches on STZ-treated animals compared to 19.6% in control). Immunohistochemical staining (Fig. 6b) confirmed expression of TRPC6 channels in the podocytes of the glomeruli and cortical tubules of the STZ-treated rats. Furthermore, it appears that expression level of TRPC6 is higher in podocytes of STZ-treated SS rats. However, it is difficult to perform quantification analysis with currently available antibodies.

## Discussion

We report here our recent findings on the role of Ang II – dependent activation of the podocytic TRPC channels in the development of DN. We have utilized the model of DN developed on the basis of Dahl SS rats^24^. The first set of experiments looked at whether 6 and 11 weeks after STZ injection were sufficient for the development of DN. As shown in Figs 1 and 2, the 11 week long treatment resulted in histological and systemic changes characteristic for DN (stable hyperglycemia, weight loss, polyuria, glomerulosclerosis, kidney tissue fibrosis, as well as microalbuminuria and nephrinuria). Basal calcium levels were increased in the podocytes of the STZ-treated rats, and patch-clamp experiments revealed that the chance of registering TRPC6 channel activity was significantly higher in the podocytes of the STZ-treated animals. These results are in accordance with the data reporting increased expression of TRPC6 channels found in the kidney cortex of subjects with acquired glomerular disease^13^,^14^,^27^. It should be noted that STZ-SS and control rats were fed a low salt diet (0.4% NaCl), and as shown before by Slaughter *et al.* both groups develop mild hypertension independent of the STZ injection^24^.

Efforts of numerous research groups have recently been focused on the role of glucose in TRPC6 regulation. It was found that in cultured podocytes high glucose induces apoptosis by stimulating TRPC6 via elevation of reactive oxygen species (ROS)^28^, which was later supported by another study demonstrating a glucose-dependent increase in TRPC6 channels expression regulated via oxidative stress^29^. Other groups reported that high glucose induced podocyte injury and subsequent apoptotic events might involve the Notch pathway (via Bcl-2 and p52)^30^, Wnt/β-catenin signaling^31^, calcineurin/NFAT2/Bax pathway^32^,^33^, or RhoA/ROCK axis^34^. Evidently, there is some discrepancy in the reported data, and the mechanism of TRPC6 channel activation in hyperglycemic conditions requires additional studies. Glucose can activate local RAS systems in the podocytes, leading both to increased production of Ang II by these cells and enhanced Ang II receptor expression^35^. Clearly, Ang II is one of the major stimuli causing calcium influx through TRPC6 channels, and there is a variety of putative pathways which could contribute to Ang II-induced podocyte injury. We have shown that Ang II causes an acute release of H_2_O_2_ in the kidney^36^, and a compelling study by Anderson *et al.* demonstrated that Ang II-dependent activation of TRPC6 channels in podocytes requires generation of ROS^37^. These findings are critical, as diabetes development is associated with oxidative stress^38^, and *in vivo* administration of the ROS scavenger TEMPOL in STZ-treated rats had a renoprotective effect and ameliorated the pathological changes in glomeruli, possibly by reducing the expression of TRPC6 channels^39^.

A recent comprehensive study by Sonneveld *et al.*^40^ conducted in cultured podocytes and STZ-treated Sprague Dawley rats elegantly demonstrated that TRPC6 channel expression is regulated by glucose in an Ang II-dependent manner. However, the type 1 diabetic STZ-treated Sprague-Dawley rat model is resistant to the development of DN^24^. The majority of recent studies on Ang II/TRPC6 interplay, although very thoroughly conducted, could have been greatly supplemented by studies carried out in an actual DN model. Here we utilized a high throughput imaging technique to monitor calcium concentration changes in the podocytes of freshly isolated glomeruli (as distinct from cultured cells), and found that Ang II – mediated calcium transient was significantly enhanced in STZ-SS rats. We have shown earlier using a mouse TRPC6 knockout model^20^ that major calcium influx in response to Ang II in the podocytes is mediated via TRPC6 channels. Furthermore, here we report the increase in the TRPC6 channels’ open probability and expression in response to Ang II in podocytes of diabetic animals. However, we do not exclude that in podocytes in diabetic condition the expression of other members of TRPC channels family could be altered as well. Furthermore, members of the TRPC family are shown to be expressed in mesangial cells^41^,^42^,^43^, and it was reported that Ang II signaling plays an important role in this type of cells^44^,^45^. Interestingly, Graham *et al.* demonstrated that abundance of TRPC6 is decreased in cultured mesangial cells treated with high glucose^46^,^47^. Furthermore, the authors showed that TRPC6 protein in glomeruli isolated from STZ-diabetic rats was decreased compared with the glomeruli from nondiabetic control rats^46^. These discrepancy with the findings reported here might be, for instance, contributed to the fact that Graham *et al.* used Sprague-Dawley rats in their experiments, and there is high possibility that in this model DN has not developed to a sufficient degree. In any case, additional studies are required to fully evaluate the role of TRPC channels in all types of glomeruli cells, including podocytes and mesangial cells.

Further delineating the mechanistic side of the Ang II-dependent regulation of the TRPC6 channels is of utmost importance in experimental nephrology, as it will provide functional tools to ameliorate proteinuria and prevent podocyte dysfunction in the early onset of DN. The use of numerous knockout rat models recently generated in the Dahl SS rat background expands our opportunities to study this mechanism. For instance, it is still unclear which Ang II receptor is involved in the signal transduction in the podocytes. Convincing data have been reported which supports the involvement of both AT_1_R and AT_2_R in glomeruli permeability to albumin^48^; both receptors are expressed in the podocytes, however the full functional relevance of this expression is yet to be revealed, particularly in disease conditions^49^,^50^.

In conclusion, we provided evidence that the effects of Ang II on calcium influx via TRPC6 in the podocytes are aggravated under the condition of type 1 DN; this finding opens up promising avenues of research to study the therapeutic modality of TRPC6 inhibition in DN or manipulating signaling pathways leading to activation of these channels.

## Methods

### Animals

Animal use and welfare adhered to the NIH Guide for the Care and Use of Laboratory Animals following a protocol reviewed and approved by the IACUC of the Medical College of Wisconsin. 12 and 18 weeks old male Dahl Salt Sensitive rats (SS/JrHsdMcwi) were used for experiments. Food (0.4% NaCl AIN-76 diet (#113755, Dyets, Bethlehem, PA)) and water were provided *ad libitum*.

### Induction of Type 1 diabetes via a streptozotocin injection

Experimental protocol used for type 1 diabetes induction in the Dahl SS rat was modified from Slaughter *et al.*^24^; a schematic illustrating the time course of the experiment is shown in Fig. 1a and Supplementary Fig. 1a (6 week long and 11 week long protocols, respectively). An intraperitoneal 75 mg/kg streptozotocin (or vehicle for control animals, 50 mM citric acid pH 4.5) injection was performed in 6 week old animals on day 0. The animals were then monitored and diabetes induction was confirmed by a blood glucose measurement (blood was obtained with a tail vein puncture) at day 5. Insulin (or sham) pellet (LinShin, Canada) was implanted *s.c.* between scapulas on day 7 in order to maintain moderate hyperglycemia (~300 mg/dL). Rats were euthanized and glomeruli were analyzed on day 42 or 77 for 6 or 11 week long protocols. Blood glucose, body weight, and 24 hour urine volume were monitored throughout the experiment.

### Kidney flush and glomeruli isolation

At the end of the protocols, the kidneys of STZ-treated or control rats were perfused (6 ml/min) through the distal aorta with saline to clear blood from the organs, then the kidneys were collected, and glomeruli were isolated according to our published protocols^20^,^51^. Minced cortical tissue was sequentially pushed through dissociation sieves of 100 and then 140 mesh (04–881-5Z and 04–881-5 X; Thermo Fisher). The suspension was then pipetted onto a 200 mesh sieve (S4145; Sigma-Aldrich) leaving the glomeruli on the top surface. The glomeruli were then rinsed into a 15 ml tube, and used for microscopy or electrophysiology experiments^26^.

### Urinary nephrin ELISA assay and biochemical analysis of protein and electrolytes in plasma and urine

Urine was collected in metabolic cages during the 6 and 11 week long experiments; urinary nephrin level was measured by a 96 well plate method with ELISA kit (Exocell, Cat #1019). Na^+^, K^+^, total protein, microalbumin and creatinine levels were analyzed as described previously^52^.

### Western blotting

Western blot analysis of the blood-free kidney cortex was performed as reported previously^52^ using antibodies against an intracellular epitope of the TRPC6 ion channel (cat no ACC-017, Alomone Labs).

### Immunohistochemistry

Isolated kidneys were fixed in 10% Formalin and routinely embedded, cut at 4 μm slices, dried and deparaffinized for subsequent histochemistry. Hematoxylin and Eosin (H&E) staining was used to assess kidney morphology. Tissue sections were incubated with anti-TRPC6 antibodies (1:50 dilution; ACC-120, Alomone Labs). Secondary detection was performed with goat anti-rabbit biotinylated IgG (Biocare) followed by streptavidin horseradish peroxidase (Biocare) and visualized with DAB (DAKO). All slides were counterstained with a Mayer hematoxylin (DAKO), dehydrated, and mounted with permanent mounting media (Sakura).

For damage analysis, the tissue was stained with Masson’s Trichrome, and individual glomeruli were evaluated using the method by Raij *et al.*^53^ and used previously by us^52^. In brief, glomeruli were scored on a 0–4 scale, where score 0 is a healthy glomerulus (no sclerosis), score 1 represented a 01–25% of mesangial expansion and sclerosis (thickening of the basement membrane and/or irregular lumina of cappilaries), 2 was characterized by a 26–50% of mesangial expansion and sclerosis (mild segmental hyalinosis involving 50% of the glomerular tuft), 3 glomeruli had a 51–75% of mesangial expansion and sclerosis (diffuse hyalinosis/sclerosis involving 50% of the glomerular tuft), and score 4 represented a 76–100% of glomerular mesangial expansion and sclerosis (diffuse glomerulosclerosis with total tuft obliteration)^24^,^54^. The tissues were randomized and coded before being submitted for blocking, sectioning, and staining. The code was not broken until all of the glomerular injury scoring was completed. Two independent trained observers performed the analysis of each of the slides before the actual rat ID was revealed for the final determination of group results.

### Electrophysiology

Cover glasses with attached glomeruli were placed into a perfusion chamber and mounted on an inverted Nikon Ti-S microscope. After a high resistance seal was obtained, cell-attached recording was performed immediately in the solutions described previously^20^,^51^. Single-channel unitary current (i) was determined from the best-fit Gaussian distribution of amplitude histograms. Activity was analyzed as NP_o_ = I/i, where I is the mean total current in a patch and i is unitary current at this voltage. P_o_ was calculated by normalizing NP_o_ for the total number of estimated channels (N) in the patch. The activity of the channels was first monitored in response to the voltage steps of 10 or 20 mV in the range of −90 mV to +60 mV in order to estimate the channel’s conductance and I-V relationship. After that, the voltage was clamped at −60 mV and the channels’ activity was recorded for several minutes before application of Ang II.

### Confocal laser-scanning fluorescence microscopy

Calcium imaging was performed with laser scanning confocal microscope system Nikon A1-R. Images were collected in time series (*xyt*, 4 s per frame) with the Nikon imaging software. Changes in intracellular Ca^2+^ concentration were estimated from fluorescence images of Fluo-4,AM (excitation at 488 nm, emission at 520 ± 20 nm). Emitted light was collected by the objective lens Plan Apo ×60 oil DIC2. The glomeruli suspension was loaded with Fluo-4,AM (5 μM; Invitrogen) dissolved in DMSO for approximately 30 min. Glomeruli were then mounted on poly-*L*-lysine covered glass in a registration chamber and washed for ~10 min with bath solution containing (in mM): 145 NaCl, 2 CaCl_2_, 4.5 KCl, 2 MgCl_2_, 10 Hepes, pH 7.35 (adjusted with NaOH). Calcium concentration measurements were conducted as described previously^20^,^55^,^56^.

### Calculations and statistics

Figure preparation and statistical analyses was carried out using MicroCal Origin software 6.0 (MicroCal Software Inc., MA, USA). Fluorescent images were processed with open source software ImageJ 1.42. All summarized data are reported as mean ± S.E.M. Statistical difference was tested with either the Student’s (two-tailed) *t*-test. Significance was accepted at *P* < 0.05 or less.

## Additional Information

**How to cite this article**: Ilatovskaya, D. V. *et al.* Podocyte injury in diabetic nephropathy: implications of angiotensin II – dependent activation of TRPC channels. *Sci. Rep.* **5**, 17637; doi: 10.1038/srep17637 (2015).

## Supplementary Material

Supplementary Information
