# Supplementary material for: Podocyte injury in diabetic nephropathy: implications of angiotensin II – dependent activation of TRPC channels
Source: Sci Rep. 2015 Dec 10;5:17637. doi: 10.1038/srep17637 (PMC4674698; doi:10.1038/srep17637)
Supplement: Supplementary Information [file srep17637-s1.pdf]

SUPPLEMENTARY MATERIAL

Podocyte Injury in Diabetic Nephropathy: Implications of Angiotensin II – Dependent Activation of TRPC Channels

Daria V. Ilatovskaya, Vladislav Levchenko, Andrea Lowing, Leonid S. Shuyskiy, Oleg Palygin, and Alexander Staruschenko

Supplementary Figure 1

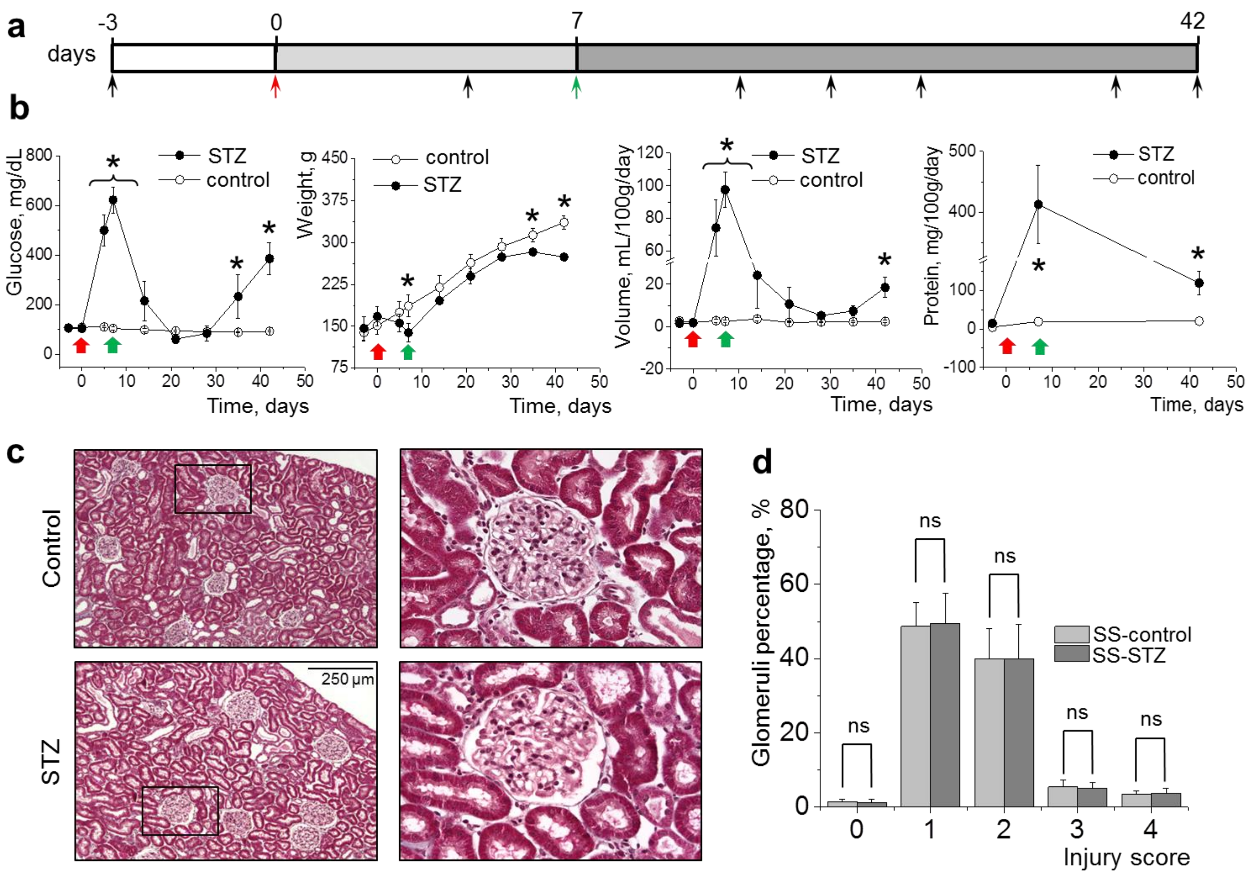

Supplementary Figure 1. (**A**) 6 week long protocol for diabetes induction. (**B**) Blood glucose, weight, urine output and protein content in control and STZ rats. (**C**) Histological characterization of the kidney. (**D**) Glomerular injury presented as a percentage of glomeruli for each score; 4 rats and 100 glomeruli per animal were evaluated per group.

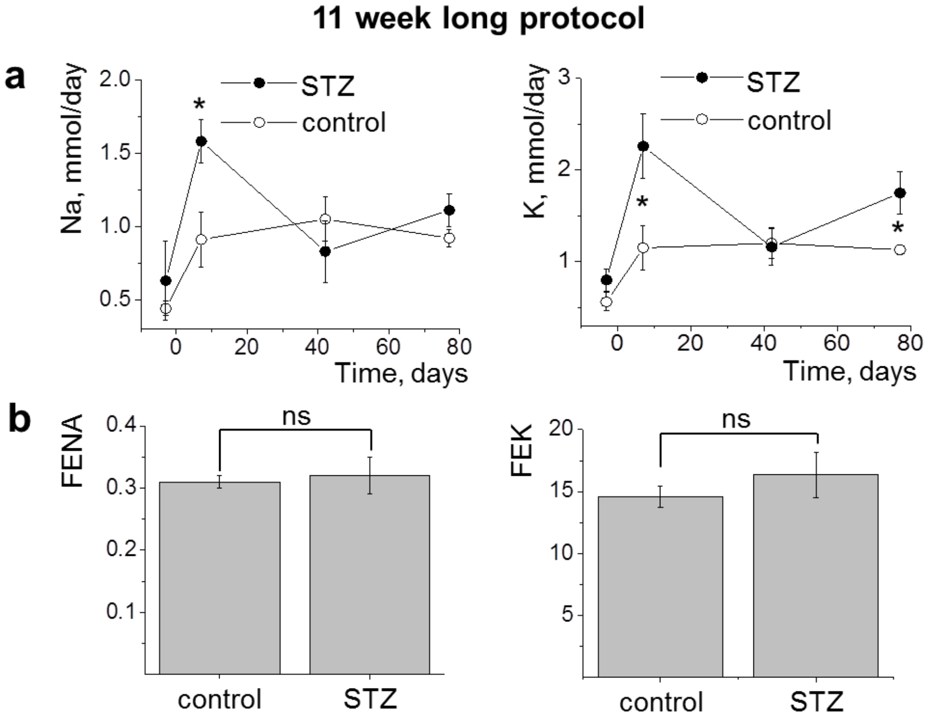

Supplementary Figure 2. (**A**) Urinary sodium and potassium excretion during the course of DN development, and fractional excretion values for sodium and potassium (**B**, terminal point, day 77). Asterisk denotes difference from the corresponding value in control rats ($p<0.05$), ns – not significant.
